# Drug Discovery of Spinal Muscular Atrophy (SMA) from the Computational Perspective: A Comprehensive Review

**DOI:** 10.3390/ijms22168962

**Published:** 2021-08-20

**Authors:** Li Chuin Chong, Gayatri Gandhi, Jian Ming Lee, Wendy Wai Yeng Yeo, Sy-Bing Choi

**Affiliations:** 1 Centre for Bioinformatics, School of Data Sciences, Perdana University, Suite 9.2, 9th Floor, Wisma Chase Perdana, Changkat Semantan, Kuala Lumpur 50490, Malaysia; lichuinchong@gmail.com (L.C.C.); 18110058@perdanauniversity.edu.my (J.M.L.); 2 Perdana University Graduate School of Medicine, Perdana University, Suite 9.2, 9th Floor, Wisma Chase Perdana, Changkat Semantan, Kuala Lumpur 50490, Malaysia; 19120005@perdanauniversity.edu.my (G.G.); wendyyeo@perdanauniversity.edu.my (W.W.Y.Y.)

**Keywords:** drug discovery, drug therapy, spinal muscular atrophy, SMA, neuromuscular disorder, computational aided drug discovery, *in silico* drug repurposing, artificial intelligence

## Abstract

Spinal muscular atrophy (SMA), one of the leading inherited causes of child mortality, is a rare neuromuscular disease arising from loss-of-function mutations of the survival motor neuron 1 (*SMN1*) gene, which encodes the SMN protein. When lacking the SMN protein in neurons, patients suffer from muscle weakness and atrophy, and in the severe cases, respiratory failure and death. Several therapeutic approaches show promise with human testing and three medications have been approved by the U.S. Food and Drug Administration (FDA) to date. Despite the shown promise of these approved therapies, there are some crucial limitations, one of the most important being the cost. The FDA-approved drugs are high-priced and are shortlisted among the most expensive treatments in the world. The price is still far beyond affordable and may serve as a burden for patients. The blooming of the biomedical data and advancement of computational approaches have opened new possibilities for SMA therapeutic development. This article highlights the present status of computationally aided approaches, including *in silico* drug repurposing, network driven drug discovery as well as artificial intelligence (AI)-assisted drug discovery, and discusses the future prospects.

## 1. Introduction

Spinal muscular atrophy (SMA) is a rare, progressive neuromuscular disease (NMD), arising from loss-of-function mutations of survival motor neuron 1 (*SMN1*) gene. It is one of the leading inherited causes of infant and early childhood mortality [1,2]. More than 95% of patients struggle from the homozygous deletion of the *SMN1* gene, which is responsible for the encoding of the SMN protein [3]. Consequently, this leads to insufficient SMN protein in neurons, resulting muscle weakness and atrophy, and in severe cases, respiratory failure and death [4]. The severity of SMA, from mild to severe, depends on the presence of the level of SMN protein [5], reflecting an inverse correlation.

Treatment options for SMA are limited and palliative in nature. Even with the remarkable results of approved drugs, the limitations, such as high cost, unknown long-term effects and side effects of the treatments, hinder the success of treating SMA patients. To date, three medications have been approved by the U.S. Food and Drug Administration (FDA) for SMA, which are nusinersen (Spinraza^®^) from Biogen, onasemnogene abeparvovec-xioi (Zolgensma^®^) from Novartis and recently approved risdiplam (Evrysdi^TM^). However, the cost of the former two therapies are astronomical in nature [6,7,8], while for the latter drug, which is in the early stage from the announcement of FDA approval, the cost has yet to be established. Besides the high cost of the treatment, the challenging drug administration for the patients with scoliosis and/or spinal deformity may require sophisticated personnel. As scoliosis is a general symptom of SMA patients, most patients do not acquire the maximal benefits from the current treatments. Several promising therapeutic approaches are currently being developed; some are at different stages of clinical trials. Despite this, the medical cost is still far beyond the affordability of the general populace.

With the advancement of computational approaches, next generation therapeutics may provide a rapid and less expensive access to new treatment. Researchers, nowadays, are gaining the advantage of computational technologies, using genomics, transcriptomics and proteomics approaches to study biological interactions that are crucial for disease pathogenesis and development of new therapies. In addition, the structural analysis on the missense mutations in SMN1 protein served a platform to understand the role of the SMN protein in SMA from the perspective of the molecular structural impact towards drug design. Furthermore, artificial intelligence (AI), machine learning (ML) and/or deep learning (DL) have shifted from hype to hope in the pharmaceutical industry due to increased research and development (R&D) cost and reduced success and efficiency rate in drug discovery. Owing to the incorporation of genomics and biochemical information, AI serves as an ‘Open Target’ platform for the prediction of therapeutic targets, which has been successfully applied to amyotrophic lateral sclerosis (ALS), one of the human neurodegenerative diseases [9]. Although there are no drug discovery studies utilizing this technology, this AI-assisted implementation may offer a future hope for SMA patients.

In this review, we provided a brief summary of the biology of SMA disease and discussed the efficacy and efficiency of currently available drugs, both approved and in clinical development. Herein, we also reviewed the past and current research that was carried out with the abovementioned computational approaches and AI-assisted drug discovery approaches in other human neurodegenerative diseases as future perspectives.

## 2. Spinal Muscular Atrophy (SMA)

SMA is a monogenic autosomal recessive genetic disorder characterized by the degeneration of alpha motor neurons (α-MNs) located in the anterior horn of the spinal cord [10,11]. The progressive destruction of α-MNs, which is responsible for initiating the muscle contraction, leads to symmetrical muscle weakness and atrophy [12,13,14]. The primary manifestations of this disease ultimately result in paralysis and often death in severe cases. Proximal muscles, specifically the lower muscles, are affected first, then the upper extremities [7,15]. SMA has a unique genetic background, as the change of functional loss in SMA peaks at the onset of the disease followed by progressive worsening condition [16].

**Figure 1:**
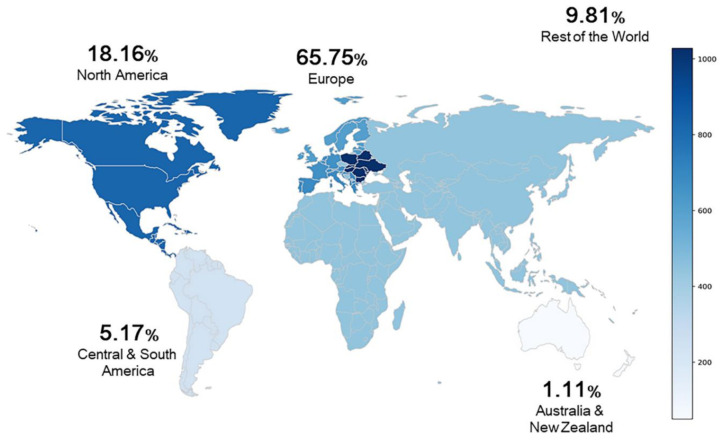
Global distribution of SMA patients who have registered under TREAT-NMD. The number of registries is rounded to two decimal places in percentage and Russia is included in the ‘Rest of the World’ herein. Data source: TREAT-NMD (https://treat-nmd.org/about-the-treat-nmd-network/ (accessed on 1 April 2020)).

SMA is the leading genetic cause of infant mortality globally [17,18] and the second most common fatal autosomal recessive disorder after cystic fibrosis [19,20]. It occurs with an estimated pan ethnic incidence of 1 in 6000–10,000 live births and a carrier frequency of 1 in 40–60 [21,22,23]. As of September 2015, a total of 4526 patients are registered under TREAT-NMD, an international network for the neuromuscular field (https://treat-nmd.org/about-the-treat-nmd-network/ (accessed on 1 April 2020)) (Figure 1). The number of SMA registries from Europe continent is generally the highest (~65.75%), especially Eastern region with 1028 patients (~22.71%). A plethora of studies suggested that there is a difference in incidence and prevalence rate between countries and ethnicities, as well as SMA subtypes [24,25,26]. A high incidence (13.7 and 17.8 per 100,000) is found from Iceland and Slovakia, countries from the European continent; however, there is a lack of details, such as the number of patients and population size, that may aggravate the interpretation of these findings [25,27,28]. The incidence of African Americans (Black) is low, although the only study concerned a Cuban population [29]. This can be explained by a lower carrier frequency among African Americans and Hispanics, as compared to Caucasians [24,26,30,31]. Notably, SMA patients can be classified into five clinical types based on age of onset and level of motor function [32].

### 2.1. Disease Etiology

**Figure 2:**
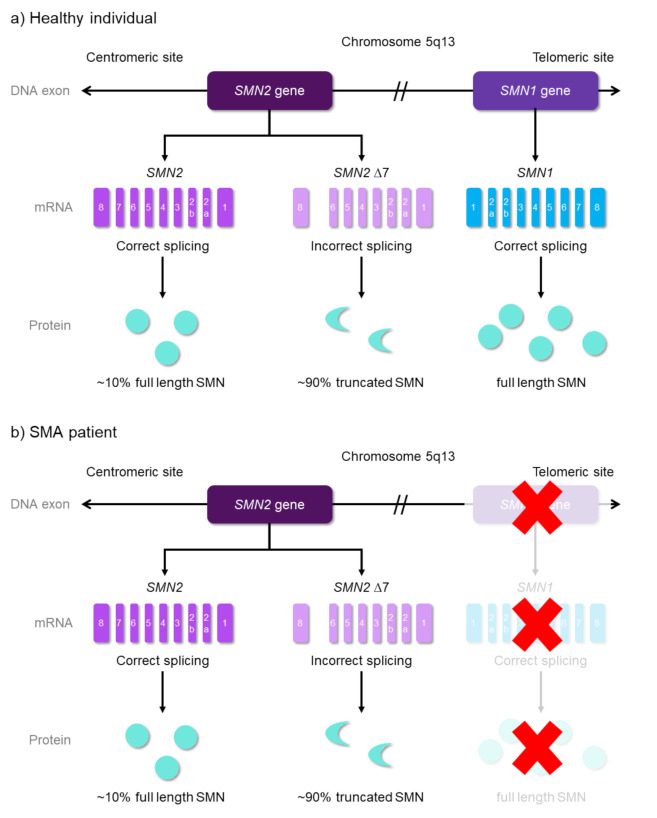
Schematic diagram of human survival motor neuron (*SMN*) gene expression for both healthy individuals and SMA patients. The telomeric *SMN1* and centromeric *SMN2* genes are identified specifically in the chromosome 5q13 region (long arm of chromosome 5). The *SMN1* gene produces all full length SMN (FL-SMN) protein, while the *SMN2* gene produces ~10% FL-SMN protein and ~90% truncated SMN protein (*SMN*Δ7) due to incorrect splicing. (**a**) In healthy individuals, both *SMN* genes are present. (**b**) In SMA patients, the absence of the *SMN1* gene, due to mutations, causes no FL-SMN protein production from *SMN1* (This condition is indicated as red ‘X’). The production solely depends on the *SMN2* gene, resulting insufficient production.

About 95% of SMA cases [33,34] are caused by a homologous deletion or mutation of the survival motor neuron 1 (*SMN1*) gene on chromosome 5q13, which is the blueprint for the SMN protein [3]. The *SMN1* is highly conserved and presents as a single copy in the genome of all eukaryotic organisms [35,36]. A normal individual has two forms of the *SMN* gene, which are telomeric *SMN1* and its paralog, centromeric *SMN2* [11,37] (Figure 2). Both genes are nearly identical, with only a difference in five base pairs. However, the base pair differences do not alter the amino acid sequence, and they encode the same SMN protein.

The *SMN1* gene produces full-length, functional SMN (FL-SMN) protein. A synonymous C-to-T base substitution (c.840C > T) at the position 6 of *SMN2* exon 7 disrupts the proper splicing and leads to a majority (~90%) of exon 7-skipped transcript (Δ7-transcript) [37,38,39,40]. Subsequent translation of such transcript results in a truncated and unstable SMN protein [17,41]. Only ~5–10% FL-SMN protein will be produced by the *SMN2* gene, whereas patients with any form of SMA lack a functioning *SMN1* gene and only depend on the *SMN2* gene. Therefore, they are in a condition of deficiency with regards to SMN protein production, and thus, they lead to a loss of motor neurons in the spinal cord.

There are five types of SMA, which are known as SMA type 0, I, II, III and IV. The copy number of *SMN2* gene modifies the severity of the disease phenotype as a high number of *SMN2* copies is related to milder phenotypes [40,42]. For instance, SMA type I patients generally have one or two *SMN2* copies, while SMA type III/IV patients have more than four copies [43,44]. Nevertheless, this inverse relationship is not always true, as a few patients with two *SMN2* copies showed milder SMA phenotypes, while there have also been patients with three *SMN2* copies that have been defined as type I [21,45,46,47]. Lacking either one *SMN* gene leads to low levels of SMN protein, though this still allows embryonic development and usually occurs in SMA carriers. Nevertheless, there are no individuals with neither *SMN* genes, which mean homologous loss, as it is hypothesized to be an embryonic lethal condition [17,48,49].

### 2.2. Clinical Classification of SMA Subtype

**Figure 3:**
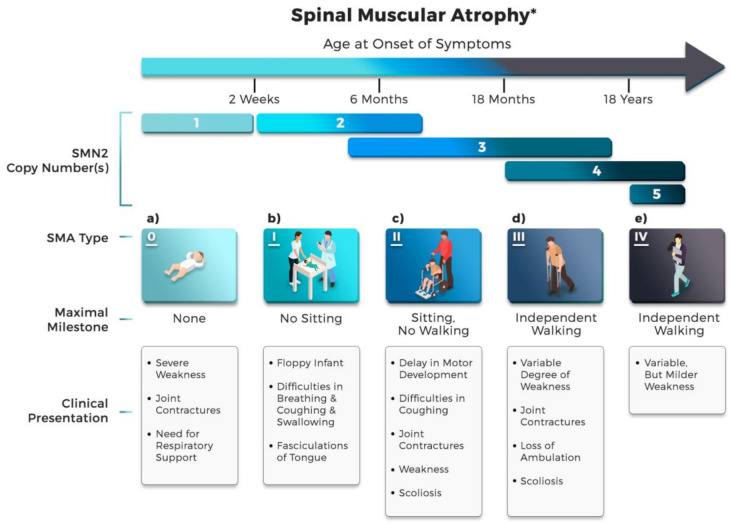
Classification of spinal muscular atrophy (SMA) sub-types. * All SMA patients, regardless of the subtypes, have no functional copies of survival motor neuron 1 (*SMN1*). SMA can be classified into five types (0-IV) ranging from the most severe form to a milder form. (**a**) Type 0 is the most severe form and in-utero onset. They normally have limited life expectancy. (**b**) Type I infants display clinical symptoms at birth or by the age of six months. They never develop the ability to sit and if no intervention is provided results in death by two years. (**c**) Type II patients are diagnosed within six to 18 months of age and they do develop the ability to sit but they never walk unaided. However, they are able to survive well into adulthood. (**d**) Type III can be further classified into IIIa (onset between 18 months to three years old) and IIIb (onset between ages of three to 30 years old). They have a normal life expectancy. (**e**) Type IV is the mildest form and adult-onset. Patients with type IV have a normal life expectancy.

The variability in severity of SMA was defined into a classification scheme in 1991 and highlighted based on the level of motor function and age of onset. There are only three SMA types in the early scheme [50]. Modifications were subsequently performed by dividing the former third category based on the age of onset, adding a Type IV as adult-onset and including a Type 0 for prenatal onset and death within weeks. Figure 3 depicts a classification of five types of SMA that are characterized by the *SMN2* copy number. Such a gene is theoretically correlated with the SMN protein level, therefore relating to the onset and severity of different subtypes of SMA.

SMA Type 0 (Figure 3a), the rarest yet the most severe form, occurs with minimal presence of the *SMN2* gene [2,51]. It is associated with an in utero onset of the affected infants with lesser movement and are often born with arthrogryposis (limited joint deformities/contractures) and hypotonia (extremely weak muscle tone, in particular respiratory and heart muscles), resulting in death before or just after birth. Some of them have respiratory failure, facial diplegia (facial paralysis) and/or heart defects, leading to death during the infancy stage [52,53,54].

Type I (Figure 3b; Phenotype MIM number from Online Mendelian Inheritance in Man (OMIM; https://www.ncbi.nlm.nih.gov/omim (accessed on 28 August 2020)), MIM 253300), the most common form of SMA (~45% of cases) [55], is also known as Werdnig–Hoffmann disease, with a severe form of muscle weakness evident at birth or within the first few months of life (~ six months at most) [2]. Most of the patients, typically present with two or three copies of the *SMN2* gene [56], have generalized muscle weakness, including an inability of controlling the movements of the head and inability to sit unaided, among others. Due to weakness of the respiratory muscles, they have breathing distress and increased the risk of aspiration [37,57,58,59]. Babies have difficulty swallowing and sucking, leading to difficulty with feeding and a failure to thrive [2].

Children of SMA Type II (Figure 3c; MIM 253550), which is also known as Dubowitz disease, have a later childhood onset between the ages of six and 18 months [2]. They are able to sit unaided; however, they are not able to stand and walk. The progressive muscle weakness worsens later in life and severely reduces life expectancy. Their symptoms are associated with scoliosis (a spine that curves side-to-side), tremors (involuntary trembling) in their fingers and respiratory muscle weakness [33,60,61]. Due to impaired bulbar function, these children develop breathing problems over time [57]. The combination of scoliosis and intercostal muscle weakness leads to respiratory insufficiency and more severely, can be life-threatening.

Approximately 30% of SMA patients are from Type III (Figure 3d; MIM 253400) [33], referred to as Kugelberg-Welander or Wohlfart-Kugelberg-Welander disease, which has an onset between 18 months to adulthood [37]. Type III is further classified into Type IIIa and IIIb, with onset between 18 months to three years old and between ages of 3 to 30 years old, respectively. Typically, patients develop a variable degree of muscle weakness, resulting in heterogeneous physical symptoms [62]. Although most of them are able to walk independently, some present with progressive proximal weakness and lose ambulation after early childhood, and the disease is usually associated with foot deformity, difficulty of climbing stairs and muscle cramps [2,33,63].

Similar to the characteristics of SMA Type III [64], patients of SMA Type IV (Figure 3e; MIM 271150) have a late onset, namely in adulthood, usually present at the age of 30 and above. This type accounts for less than 5% [33] of overall SMA cases, and hence, is considered as a mild form of SMA. Comparatively, they might have minor disabilities; nonetheless, they are able to achieve motor milestones and have normal life expectancy [2,62].

### 2.3. SMN Protein

**Figure 4:**
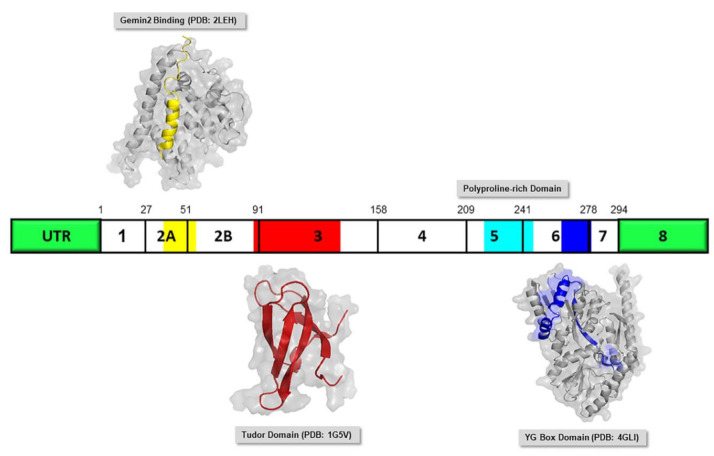
Diagrammatic representation of the full length SMN (FL-SMN) protein with its respective available protein structure. The number of exons is indicated within the boxes of SMN protein diagram with the number of the last amino acid residue of each exon, indicated above. The PDB structure of the domains are illustrated in the same color that overlaid in the SMN protein diagram indicating the location of the respective domains (Gemin2 Binding: yellow; Tudor Domain: red; Polyproline-rich Domain: cyan; YG Box Domain: blue). Abbreviation: UTR, untranslated region.

Mutation events of the *SMN1* gene that encodes the SMN protein is predominantly linked to SMA disease [65]. Understanding the molecular structural of SMN protein is important and helpful in molecular pathogenesis of SMA. However, as of January 2021, there is still no FL-SMN protein structure in Protein Data Bank (PDB; https://www.rcsb.org/ (accessed on 29 January 2021)) and only have SMN-related structures. The SMN, a 38-kDa protein, is ubiquitously expressed in both nucleus and cytoplasm [66] in particular, having a high concentration in motor neurons of the spinal cord and relatively less in lymphocytes and fibroblasts [67]. Human SMN protein is coded by eight exons [68] and it consists of 294 amino acids and harbors several functional domains, including a basic/lysine (K)-rich region, a Tudor domain, a proline (P)-rich region and a tyrosine-glycine rich (YG)-box (Figure 4). Those functional domains are highly conserved from yeast to human and play important roles in the motor system as well as intracellular processes [69].

The Gemin2 binding domain (Ge2BD), coded by exon 2 and located near the N-terminus, is highly conserved among SMN-containing eukaryotes, suggesting the important role of SMN–Gemin2 interaction [70,71]. Gemin2 is a core protein that functions in the formation of SMN complex and also aids in the spliceosomal small nuclear ribonucleoprotein (snRNP) assembly via the stabilization of the SMN complex [72]. The p53, a tumor suppressor protein and also transcription regulator, interacts with the domain coded by exon 2 [73]. The association of p53 with SMN suggests the likelihood for apoptosis to occur. However, when this interaction is reduced, this could lead to death of motor neurons, as shown in SMA.

A central Tudor domain, coded by exon 3 of SMN, facilitates the protein-protein interactions [74]. The domain, which is usually found in RNA-associated proteins, recognizes symmetric dimethylarginine (sDMA) modifications in arginine/glycine rich regions (RG domains) of proteins, including Sm proteins (B, D1 and D3) [70,75,76,77]. Interestingly, SMA-causing mutations of this domain impair sDMA peptide binding [70]. The proline-rich region, coded by exon 4 to 6, interacts with prophilin, a key protein in controlling the actin dynamics in the cells [78,79].

A most conserved segment near the C-terminus [71], referred to as the ‘YG-box,’ is responsible for oligomerization, which is crucial for the function of SMN and interaction between SMN with Gemins and Sm proteins. It is also involved in the interaction with Gemin3 (a dead-box helicase) [80], ZPR1 (a zinc-finger protein) [81] and SIN3A (a transcription co-repressor) [82].

**Figure 5:**
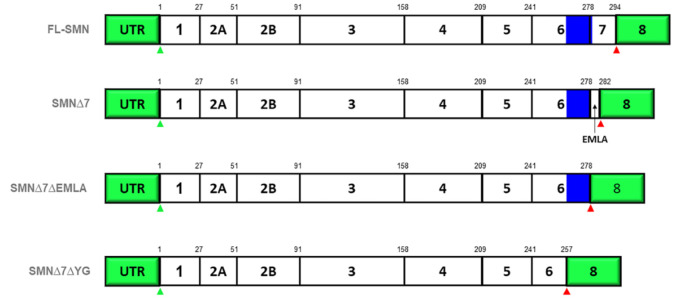
Schematic diagram of transcripts generated from SMN with a series of deletions. The name of the transcript is shown on the left, which indicates the deleted region. For instance, Δ7 refers to the deletion of exon 7. The start and stop codons are depicted in green and red triangle icons, respectively. The YG region, a conserved tyrosine/glycine rich motif, in exon 6 and 7 is indicated by the blue box while EMLA, a four-amino acids motif that replaced the 16-amino acids of exon 7, is indicated with an arrow. Abbreviation: UTR, untranslated region.

Given the SMN protein’s critical role in the biogenesis of snRNPs, SMA patients fully depend on *SMN2* gene to compensate the loss of the *SMN1* gene for the production of the SMN protein. However, a relatively low amount of functional SMN protein is produced while the translated product of aberrant splicing event, termed SMNΔ7 (only consists of 282 residues; Figure 5), is unstable and rapidly degrades [83]. The half-life of a functional SMN is >8 h, while SMNΔ7 is about 3 h. A study suggested that the addition of a four-amino acids motif, EMLA, and a conserved tyrosine/glycine rich motif, YG region, at the C-terminal, reduce the stability of SMNΔ7 [71,83]. EMLA serves as a degradation signal for Δ7-transcript, is coded by exon 8, while the YG region is coded by exon 6 and 7 [71]. Interestingly, the future deletion of EMLA and YG region from SMNΔ7 alone, termed as SMNΔ7ΔEMLA and SMNΔ7ΔYG, respectively (Figure 5), show the increment of the half-life [83].

## 3. Current Drug of SMA

It is well known that the disease severity is related to the SMN protein levels, and thus, increasing SMN production has been a major SMA drug discovery strategy [84]. Multiple mechanisms have been targeted to drive higher expression of the full length SMN protein, either from the *SMN2* gene or from the exogenously restored *SMN1* gene [35,62]. The SMN protein was suggested to play a crucial role in neurons and muscle [85,86,87]; hence, SMN-independent therapies that provide neuroprotection or slowing down or halting the events due to the effects of SMN depletion could be an alternative for SMA [88].

### 3.1. Current Drug—Early Success

Out of 1167 US Food and Drug Administration (FDA)-approved drugs (as of March 2020), there are only three drugs approved for the treatment of SMA, which are nusinersen, onasemnogene abeparvovec and risdiplam.

**Figure 6:**
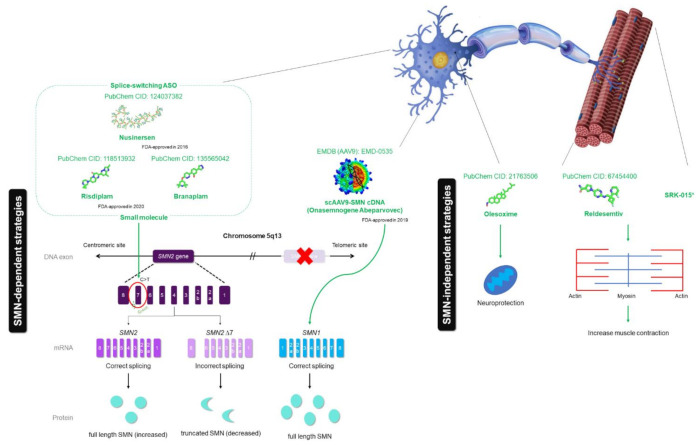
Therapeutic mechanism of SMA drugs, including three FDA-approved drugs (nusinersen, onasemnogene abeparvovec and the recent FDA-approved drug risdiplam) and drugs that are in clinical trials (branaplam, olesoxime, reldesemtiv and SRK-015). Nusinersen (PubChem CID: 124037382), a synthetic antisense oligonucleotide (ASO), is designed to hybridize intronic splicing silencer N1 (ISS-N1), which is heterogenous nuclear ribonucleoprotein (hnRNP) A1-dependent, to facilitate accurate splicing of *SMN2* transcripts. Onasemnogene abeparvovec (no available structure) is a gene therapy that targets the *SMN1* gene replacement using adenovirus vector AAV9 (EMDB: EMD-0535). Risdiplam (PubChem CID: 118513932) and branaplam (PubChem CID: 135565042) are small molecules that have the same mechanism of action as nusinersen. The red ‘X’ mark represents the deleted *SMN1* gene. Other than SMN-dependent drugs, olesoxime (PubChem CID: 21763506) acts as neuroprotective compound, while reldesemtiv (PubChem CID: 67454400) and SRK-015 act as a fast skeletal muscle troponin activator (FSTA) and myostatin inhibitor, respectively, to increase the muscle contraction.

Nusinersen (Trade name: Spinraza^®^) was the first therapy approved in late 2016 by the FDA to treat this rare NMD [89]. Nusinersen, a modified 2′-*O*-methoxyethyl phosphorothioate antisense oligonucleotide (ASO), effectively modulates the splicing of SMN transcripts [6,90,91,92]. The drug is directly administered to the central nervous system (CNS) via intrathecal injection to modify the splicing process of *SMN2* pre-mRNA by promoting exon 7 retention, resulting in the enhancement of the FL-SMN protein expression level [90,93] (Figure 6). Hua et al. (2010) highlighted that nusinersen provides phenotypic and pathologic benefit in the animal models, both mild and severe SMA through direct injection into CNS. This was in agreement with Passini et al. (2011), who worked on improving the efficacy of ASO [94]. However, due to the inability of the transverse of the blood–brain barrier, it is applied via injection into the spinal canal for SMA therapeutic application [95].The intrathecal route allows the direct delivery of drugs to CNS by circumventing the blood–brain barrier [96]. The recommended dose for nusinersen is 12 mg [6]. Results on the trials on nusinersen suggested that some patients can even achieve certain milestones that have been lost without treatment, including sitting, standing and walking. This treatment has been suggested to be given early in the course of the disease and worked efficiently among the patients with SMA type I, II and III [97]. The major drawback of this treatment is that the patient has an increased risk of getting upper and lower respiratory tract infections and constipation [98].

In 2019, onasemnogene abeparvovec (onasemnogene abeparvovec-xioi; formerly AVXS-101), under the trade name Zolgensma^®^ has been approved by FDA as the second disease modifying SMA treatment for patients aged up to 2 years old with SMA type I [99]. It comprises the capsid of adeno-associated virus 9 (AAV9), delivering complementary DNA (cDNA), which codes for the SMN protein, to its target motor neurons [100,101]. With a single, one-time intravenous (IV) administration, AVV9 crosses the blood–brain barrier and delivers a working copy of the *SMN1* gene able to reach patients’ cells, allowing the production of the SMN protein (Figure 6). Additionally, the *SMN1* transgene, along with the synthetic promoter that consists of the AVV9, plays an important role to sustain SMN protein production in the long term. Given its effectiveness in resolving the SMA molecular defect, it adversely affects the liver by increasing the level of serum aminotransferase, a liver enzyme [7,101,102]. However, elevated liver enzymes can be controlled by using prednisone [16]. Hence, patients need to be monitored for their liver function at least three months after administration [7].

Recently (as of 7 August 2020), risdiplam (Trade name: Evrysdi^TM^) was approved by the FDA as the first oral drug for SMA patients [103]. Risdiplam, formerly known as RG7916, is an investigative drug being developed by Hoffmann-La Roche in collaboration with PTC Therapeutics and SMA Foundation to treat all types of SMA [62]. Similarly to nusinersen, it acts as a *SMN2* splicing modulator (Figure 6) and improves the efficiency of the transcription of the *SMN2* gene, thus increasing the systemic SMN protein concentration. Ratni et al. (2018) demonstrated the two-fold increment in SMN protein concentration after 12 weeks of therapy in patients [104]. However, it may cause some side effects, including fever, diarrhea, rash, upper respiratory tract infections, pneumonia, constipation and vomiting according to the respective clinical trial (FIREFISH for infants aged 2 to 7 months and SUNFISH for children and adults aged 2 to 25 years) [103].

Despite the discovery of promising therapeutic strategies, the limitations, including the treatment viability (in the case of nusinersen), long-term effects, side effects and cost, among others, are highlighted. As the drugs need to pass through the blood–brain barrier (BBB), nusinersen must be administrated locally through an intrathecal injection. This route of administration is challenging and requires sophisticated personnel and technique, such as image-guided technique, particularly for patients with scoliosis and/or spinal deformity [105]. Moreover, elevated costs of nusinersen (~USD $125,000 per injection) associated with screening and subsequent treatment (~USD $750,000 in the first year and ~USD $375,000 annually for subsequent year) place this drug among the most expensive drugs [6,106]. For the latest approved gene therapy, onasemnogene abeparvovec costs ~USD $2.125 million per injection, although only a single treatment is required for each SMA type I patient [107], while the cost of risdiplam (the most recent FDA-approved drug) is yet unknow. Additionally, as all are relatively new therapies, there are no longitudinal studies for long-term effects, although there is a plethora of studies for side effects. Therefore, a more cost-effective drug with an alternative route of administration is required for this devastating SMA.

### 3.2. Existing Drug—Clinical Trial Stage

Several therapies (Table 1) aiming to increase SMN protein level have been studied with a different approach, which is small molecule-based. With the promising preclinical results, risdiplam and branaplam are currently being tested in clinical trials. Risdiplam (Table 1), the recent FDA-approved drug, is still being evaluated in two clinical trial programs, which are JEWELFISH (ClinicalTrials.gov identifier: NCT03032172) and RAINBOWFISH (ClinicalTrials.gov identifier: NCT03779334) [108]. The former trial is aiming for SMA patients aged 6 months to 60 years old, while the latter trial is for those infants from birth to six weeks who are asymptomatic but genetically diagnosed with SMA.

Branaplam (Table 1), developed by Novartis Pharmaceuticals, also known as LMI070 and NVS-SM1, acts similarly to risdiplam to improve the SMN protein concentration by correcting the splicing defect in human *SMN2* gene [62,109]. As the clinical trial phase II (ClinicalTrials.gov identifier: NCT02268552) only began in July 2019, there is a lack of information regarding this therapy.

Although there are some promising clinical and preclinical results from ASOs, small molecules and gene therapies on *SMN2*, there are still some negative impacts on efficacy [100]. Hence, with the specific function of SMN protein by involving in the neuronal actin cytoskeleton [110], expanding the repertoire of targets, for example drugs to improve neuromuscular function, is an alternative for SMA drug discovery to complement the efficiency.

In collaboration with Astellas, reldesemtiv (Table 1), formerly known as CK-2127107, is being developed by Cytokinetics. It acts as a troponin stimulant that may improve muscle mass and function in SMA and amyotrophic lateral sclerosis (ALS) patients [62,111,112]. This drug slows down the release of calcium from the regulatory troponin complex of fast skeletal muscle fibers. The sarcomere is then sensitized to calcium, leading to improved skeletal muscle contractility and physical performance in a human cohort [113]. Notably, the interim analyses of SMA patients showed mild improvement for six-minute walk test (6MWT) and maximal expiratory pressure (MEP) [23]. With the positive result of Phase I, reldesemtiv is now under investigation in Phase II clinical trial (ClinicalTrials.gov identifier: NCT02644668) [111].

Developed by Scholar Rock, SRK-015 (Table 1) is a biological monoclonal antibody against myostatin [23,62]. Myostatin, primarily found in skeletal muscle cells in latent form, plays an important role in inhibiting the muscle growth and maintaining the skeletal muscle mass [114]. With this mechanism of action of SRK-015, muscle tissue of SMA patients is plausible to convert into active form. The positive result in Phase I (well dose tolerance up to 30 mg/kg) provides opportunities for patients with SMA Type II and III for Phase II (TOPAZ; ClinicalTrials.gov identifier: NCT03921528).

## 4. Computer-Aided Drug Design (CADD)—The Open Window of Therapeutic Agents

With technological advances in the areas of molecular structure characterization, computational science and molecular biology, CADD is a promising avenue to facilitate the discovery, design and optimization of potential therapeutic agents in the era of big data. Not only does it reduce the time for drug discovery, CADD plays a prominent role in reducing the quantity of testing molecules in vitro or in vivo [115,116]. By predicting the numerous small molecules, either natural or synthetic compounds, that bind favorably to the target macromolecules, the number of trial experiments can be minimized.

Of neurological disorders, the discovery of efficient CNS drugs is more challenging as compared with other diseases [117]. There are several challenges, in general, throughout the drug discovery process. The most notable obstacle, in the process of lead optimization, is due to the presence of the blood–brain barrier that restricts the flow of molecules to the brain. Nonetheless, it is possible to overcome and predict biological activity, pharmacokinetics (absorption, distribution, metabolism and extraction; ADME) as well as toxicity with the advent of more sophisticated computational approach such as the high throughput screening (HTS) method and CNS multiparameter optimization algorithm [117,118].

Approved or investigated drugs, either SMN-dependent or SMN-independent, were identified with an impressive preclinical or clinical effect; however, none of them are able to cure the disease alone. Hence, this invokes the compelling motivation to implement a CADD approach to speed up the development of the SMA drug—*in silico* drug repurposing, network-driven drug discovery (NDD) and artificial intelligence (AI)-assisted drug discovery (AID).

### 4.1. In Silico Drug Repurposing

Drug repurposing, also known as drug repositioning, is one of the emerging potential approaches to circumvent the cost and time required for the development of an efficacious treatment [116,119]. It is defined as a process of identifying new therapeutic indications for an approved drug. Recently, with the encouragement of fast track marketing authorization procedure (FDA approvals), this approach has been widely used for rare diseases [119], including SMA [120], because it offers several benefits over the classical de novo development process of drugs. The approved drug compounds, in essence, have passed safety efficacy, allowing an omission of Phase I clinical trials [120,121].

Several studies have successfully repurposed FDA-approved drugs for SMA treatment and showed plausible in vitro activities, such as enhancing the SMN2 promoter activity, modulating SMN2 splicing and stabilizing *SMN2* mRNA or SMN protein [62,112,120]. Histone deacetylase inhibitors (HDAC), including sodium butyrate, phenylbutyrate and valproic acid (VPA), among others, to date, have been explored with SMN2 promoter activity [112,122,123,124,125]. They have demonstrated an increase of SMN protein levels in patient-derived cells as well as in animal models. HDAC induces the alteration in the chromatin structure into a tight-coiled transcriptionally-repressed region of chromatin, thereby activating the gene expression [112,126]. The ability to reverse the cell transcription held some promise on SMA. Notably, a plethora of studies demonstrated that the most promising HDAC, VPA, increases two- to four-fold of full length SMN protein in patient-derived fibroblast cell lines [125,127]. A recent study by Pagliarini et al. (2020) suggests that the combination of HDAC and nusinersen exerted synergistic effect in enhancing the expression of SMN2-derived FL-SMN protein [128]. This may reduce the frequency of nusinersen administration, leading to a reduction of the financial burden for SMA patients.

In essence, SMN-independent drugs are centered on neuroprotective and muscle enhancing approaches. Neuroprotective drugs aim to improve the motor neuron function while muscle enhancers aim to increase the muscle mass and enhance the muscle contractibility [111]. In referencing to the localization of the SMN protein in neuronal cells, neuroprotective drugs for other CNS diseases could be a better option to reposition for preventing and/or delaying motor neuron death in SMA. Approved neuroprotective drugs, such as riluzole, hydroxyurea and rasalgiline, which modulate regulatory pathways in CNS, may be an option for SMA therapy [62,112,120]. For instance, riluzole, an approved drug for ALS, exhibits neuroprotective effects through glutamate reduction. The interim analyses in SMA animal model showed stabilization of neuromuscular junctions; however, it failed to yield the promising clinical trial result [111]. This may be due to poor pharmacokinetics properties, leading to poor long-term efficacy [129]. Despite the limited successes of riluzole, repurposing FDA-approved drugs for CNS disease, including rasalgiline, is an interesting avenue for SMA.

Given the potential of the drug repurposing approach, with the combination of publicly available databases and computational methods, the *in silico*-based approach may provide benefits, in terms of time and cost, towards the drug discovery process by narrowing down the top hits through *in silico* validations [130]. Public repositories for relevant experimental and biological data, including chemical structures, gene expression, drug disease association, phenotypic traits, side effects and more, are treasure troves for *in silico* drug repurposing. Few important databases that are widely used in drug repurposing studies are collectively outlined in Table 2 with corresponding URLs. Owing to the wealth of multi-omics data, different methods have been adopted in drug repurposing, which can be divided into two major categories: (i) drug-oriented and (ii) disease/therapy-oriented [131].

Drug-oriented drug repurposing strategies require the knowledge of cheminformatics and bioinformatics as foundation, including drug information, chemical structures of drug and target, drug-target network, signaling or metabolic pathway and genomic information. Information on chemical structure of small molecule compounds can be easily retrieved from the widely used chemical structure database, such as PubChem [155], ChEMBL [137] and DrugBank [145], among others. The RCSB Protein Data Bank (PDB) [156] is the primary database for the three-dimensional (3D) structure of protein target. This 3D-structure information is crucial for structure-based screening, which aims to reveal how a ligand binds to the protein target with the aid of molecular docking. Alternatively, a structure information can be codified into line notation—Simplified Molecular Input Line Entry System (SMILES) [186] and International Chemical Identifier (InChl) [187] that can be easily analyzed algorithmically. Moreover, the chemical structure similarity approach between ligands suggests two molecules that have similar structure are likely have similar bioactivities [188]. This can be measured using chemical structure fingerprints, either two- (2D) or three-dimensional (3D), or binary, with a distance metric such as Euclidean, Manhattan and Mahalanobis (in the case of non-binary chemical fingerprints) as well as Tanimoto coefficient (Tc; in the case of binary chemical fingerprints) [189,190]. Incorporated with drug-target interactions (DTIs), the chemical structure similarity between drug compounds and ligand targets may reveal unforeseen associations. DTIs, which are available in BindingDB [133], ChEMBL [137] and DrugBank [145], among others, can simply indicate the presence or absence of an interaction. This binary-level information is useful to employ in pharmacophore modeling, and few models have been recently developed [191,192,193]. Other than chemical structure data resources, genomics data that are available from the National Center for Biotechnology Information (NCBI) GenBank, Gene Expression Omnibus (GEO) [172], Single Nucleotide Polymorphism database (dbSNP) [175] and Sequence Read Archive (SRA) [174] are also important to understand the disease and drug mechanism of actions in order to provide insight on the discovery of new uses for existing drugs.

The disease-oriented approach is only applicable if the information of disease model is available and commonly used to study the contribution of pharmacological characteristics towards drug repositioning effort on a particular disease. Incorporated with clinical trial information, for adverse drug events (ADEs) and FDA approval labels that are available from ClinicalTrials (https://clinicaltrials.gov/ (accessed on 28 August 2020)), Drug@FDA (https://www.accessdata.fda.gov/scripts/cder/daf/ (accessed on 28 August 2020)), side effect resource (SIDER) [183] and more, this strategy shows promise to identify off-target effects and predict the side effects and ADEs of drugs, leading to the improvement of the efficiency of drug discovery. As of December 2020, SIDER (version 4.1), a comprehensive side effects database, comprises 1430 drugs with 5868 side effects, resulting in 139,756 drug–side effect pairs [183]. This pair information can be applied as features in building a prediction model for disease indications, which has been implemented by Yang and Agarwal (2011) [194], Bisgin et al. (2014) [195], Ye et al. (2014) [196] and Sridhar et al. (2016) [197]. Compared with the drug-oriented approach, this requires more specific knowledge of drug and disease, including the gene signatures and disease pathways. Gene signatures, defined here as the sets of significantly up- and down-regulated genes, derived from disease omics data, are publicly available from the connectivity map (CMap) [140,141] and the National Institutes of Health (NIH) library of integrated network based cellular signatures (LINCS) [151]. The advantage of integrating this information in drug repurposing is the added involvement of molecular- and/or genetic-level mechanisms, leading to the discovery of hidden mechanisms of drug and target [198]. The disease-specific pathway-based approach utilizes metabolic and signaling pathways, gene expression correlation and protein interaction network information to narrow down the target proteins/molecules from a general signaling networks to a specific network and predict the connection between drugs and disease. Notably, Kyoto Encyclopedia of Genes and Genomes (KEGG) [163,164] is a frequently used database for such approach. The network is a way to discover informative relationships between drugs and targets that consists of two main entities, which are nodes and edges. The nodes in such network are represented as genes, proteins, molecules or other biological entities, while the edges are the connections that can be weighted based on the attributed information.

The blooming of drug repurposing resources and the advances in computational sciences give rise to the development of novel algorithms/tools and approaches that are capable of capitalizing on publicly available data. A list of widely used drug repurposing approaches is summarized in Table 3 with their respective required data and software tools, though the table is neither extensive nor exhaustive.

Although more databases are increasingly being established with enormous information, drug repositioning still remains as a tremendous challenge, especially for rare diseases, as drug repurposing studies are highly dependent on the available information and knowledge on disease mechanism, target protein/gene. Moreover, there are numerous available drug repurposing methods based on the availability of specific information. Hence, choosing the proper approaches and tools to mine novel knowledge based on the study of interest is extremely crucial, as otherwise the success of this approach may be hindered. It is necessary to emphasize the importance of integration of computational and experimental methods, and in-depth mechanistic computational pipelines or models in order to maximize the success rates of drug repurposing.

### 4.2. Network-Driven Drug Discovery (NDD)

Network biology epitomizes the cell as a cluster of molecules interacting with one another and aims to illustrate the emergence of cellular phenotype from the network of molecular interactions [227]. The networks can be regarded as establishing the mechanistic bridge between the constituent molecules of a cell and the phenotypes that the cells demonstrate. This perspective alone considers the cellular mechanism of disease to be materialized due to networks of pathological interactions that occur only in the disease state. In this context, drug discovery can, hence, be perceived as the search for agents that significantly disrupt these pathological networks. NDD, as a whole, aims to identify signatures of molecular perturbations; that is, collections of multiple proteins, that significantly disturb the structural integrity of the cellular networks bringing forth the targeted disease mechanism [228]. The search space of therapeutics, such as small molecules, biologics or other agents, can then be screened and narrowed down based on their ability to produce the identified perturbation signature. It should be acknowledged that the compounds of this scheme are not expected to directly bind to all proteins within the identified signature, but rather to produce a downstream, functional effect on the molecules making up the signature [229]. This approach is far removed from the traditional target-driven drug discovery that focuses on specific drug targets, whose downstream effects will significantly perturb the disease phenotype without much emphasis on cellular networks for understanding the underlying disease mechanisms.

**Figure 7:**
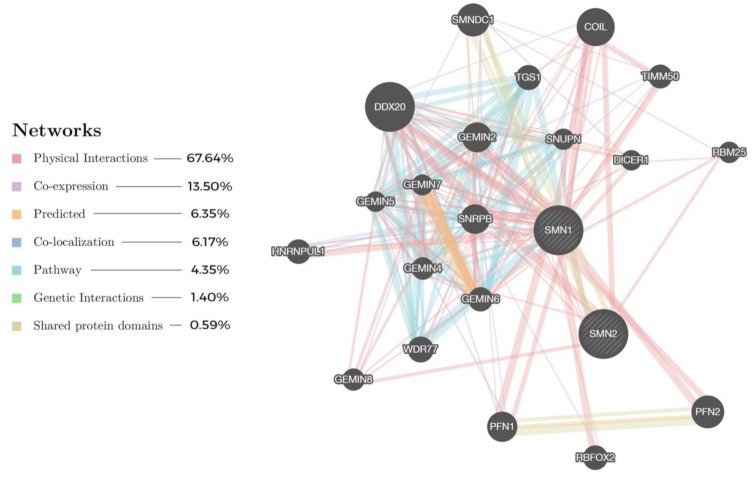
Protein network based on two main proteins, SMN1 and SMN2, and their respective interactions with other proteins related to SMA, generated using GeneMANIA [223] (https://genemania.org/ (accessed on 29 December 2020)). The most prevalent network relationship, reported by literature, among the proteins is physical interactions (pink color) at 67.64%, as visually shown by the line thickness, while the smallest belong to the shared protein domains at 0.59%.

As opposed to the canonical SMN-independent treatment based on many disease-modifying pathways, potential drug targets may be found on the periphery of the pathways using the NDD approach. A network analysis based on the two main proteins (Figure 7), SMN1 and SMN2, as protein input in GeneMANIA (https://genemania.org/ (accessed on 29 December 2020)) [223], has generated a network of putative interacting proteins that works in unison to bring about the phenotypes as seen in SMA. Proteins such as GEMINs [230], SNRPB [231], DDX20 [232] and PFN2 [233] appear to be highly correlated to the functioning of SMN1 and SMN2. These proteins are essential to SMN in forming macromolecular complexes (e.g., SMN-GEMINs, SMN-snRNPs) to chaperon the assembly of small nuclear ribonucleoproteins (snRNPs) that are vital to pre-mRNA splicing for producing the final SMN1 and SMN2 proteins [230]. Modulating these proteins in the cellular network within the context of SMA may serve as an opportunity to develop novel therapeutics complementary to the conventional SMN-dependent treatments in addressing the challenge of creating a robust and sustainable solution to curing SMA.

With the advances of network biology, the rapid growth of publicly available biomedical data and the advanced computational analytics, the NDD approach, a mechanistic based approach, proposes an alternative to identify the novel target as potential SMN-independent treatment. Collectively, a comprehensive analysis of drug-protein interactions on a genome-wide scale is crucial and provides beneficial effects in drug discovery, especially for polypharmacology and phenotypic screening [234]. Several studies has discovered many disease-modifying pathways in SMA, such as the RhoA/Rho kinase (ROCK) [79,235,236], the cyclic adenosine monophosphate (cAMP) pathway [237], the extracellular regulated kinase (ERK) [235,238], the c-Jun N-terminal Kinase (JNK) [239] and the p53-pathway [240], which show promise as further SMA therapy development [100]. For instance, Y-27632 and fasudil, ROCK inhibitors, have been suggested to improve the lifespan of an intermediate SMA mouse model (Smn^2B/-^) without any effects on the expression levels of the SMN protein [236,241,242]. Discussing all the listed signaling pathways in detail would go beyond the scope of this review. Nonetheless, with the given example, it is clear that a more in-depth level of understanding those pathways is likely to provide further insights in identifying novel therapeutic targets in a much shorter period.

### 4.3. AI-Assisted Drug Discovery (AID)

**Figure 8:**
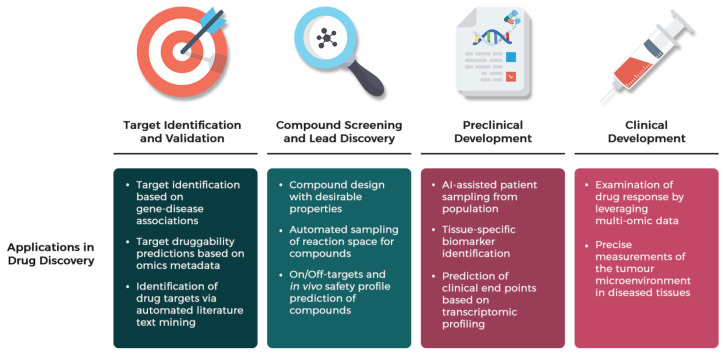
Machine learning applications in the drug discovery pipeline. Promising developments of pioneering ML research has brought forth unprecedented advances across various stages of the traditional drug development pipeline, especially the concept of automation in the early drug discovery process of target identification and validation & compound screening and lead discovery; relying on the domain of NLP in AI to find prospective drug targets by scanning upon thousands of relevant literature based on contextual information in research papers, and integrating AI with synthesis robots to explore unknown reaction space to search for drug candidates in which multiple chemical experiments are conducted automatically in real-time to assess the reproducibility of chemical reactions and discover new reaction outcomes. AI in the preclinical development has been a game-changer for patient selection in Phase II and III clinical trials by identifying and predicting human-relevant biomarkers of diseases, thus preventing unnecessary toxicities and side effects of consuming the experimental drugs for the designated patients [254].

AI application to drug discovery is not a new technology and started around 1990 [243,244,245]. Driven by the big data in the field of biomedical and/or healthcare, the advancement of algorithms and technology such as deep learning (DL), graphical processing units (GPUs) and Google’s tensor processing units (TPUs) enable better predictive capability by shortening the computing time [246,247]. To date, AI has been extensively adopted to support healthcare services and research. Virtual screening [248], quantitative structure-activity relationship (QSAR) [249], de novo drug design [250,251], drug repurposing [252] and chemical space visualization [253] utilized ML extensively to reduce the gap in the conventional methods in drug discovery, while DL shows promise in proposing potent drug candidates using their properties and toxicity risks [9]. Uptake from the pharmaceutical industry is still lagged, especially for rare diseases. Given the breadth of AID, we summarized the pipeline and its pre-requisites (Figure 8).

To date, there are only a few drugs that utilized AID that are being conducted for clinical trials; nonetheless, none of them have proceeded to Phase III and above. DSP-1181, reportedly the first AID-designed drug in January 2020, which has begun with human testing, was developed by Exscientia with Japan’s Sumitomo Dainippon Pharma [255] with the intention of treating obsessive compulsive disorder (OCD) patients. Additionally, to date, there are no studies utilizing AI for the drug development of SMA; however, only some case studies of AI being implemented to tackle one of the rare diseases that closely related to SMA, which is amyotrophic lateral sclerosis (ALS). The breakthroughs from BenevolentAI and Verge Genomics have demonstrated promises for therapeutic approaches in ALS by leveraging the AI technology [256,257]. A plethora of studies have successfully implemented ML models in ALS research, commonly with random forests (RF) [258], support vector machines (SVM) [259], neural networks (NN) [260] and more. Chiefly, although all case studies related to ALS are still being evaluated, these studies may serve as a proxy, so we can extrapolate the efficacy of AI methods employed by others in developing drugs to treat SMA.

Through a closer inspection of AI techniques in accelerating drug discovery, there are several common machine learning methods being employed to address the challenges in two major areas of drug development: (i) design and discovery and preclinical research; and (ii) clinical research and safety monitoring (Table 4). In the first major area, a generative model has been utilized for de novo drug design, as reported by a study conducted by Prykhodko et al. (2019), who proposed a novel deep learning architecture, LatentGAN, which combines an autoencoder and a generative adversarial neural network (GAN) to generate novel structures [261]. The autoencoder was first pre-trained on one-hot encoded SMILES data derived from ChEMBL database for mapping structures to latent vectors. These outputs are then fed into the GAN architecture as training data to generate novel latent vectors that were later decoded in the autoencoder to obtain the SMILES strings of the novel molecule. It was found that the LatentGAN was able to generate similar drug-like compounds after training on a randomly selected 100,000 ChEMBL subset data when compared to the 200,000 generated compounds from LatentGAN in a 2D PCA plot (explained variance 74.1%) to examine the coverage of the chemical space. A similar study was carried out by Kadurin et al. (2017) in investigating the viability of utilizing GANs and autoencoders to generate new molecules with desired molecular properties *in silico* [262].

In the second major area of clinical research and safety monitoring, deep representational learning was used in a novel architecture, DeepEnroll, to streamline the process of finding qualified patients for clinical trials with an NLP-based model called Bidirectional Encoder Representations from Transformers (BERT), which utilized heterogeneous data from EC (Text Data) and patient EHR (Tabular Data) to train the model and optimize the patient-trial matching score in a cross-modal inference fashion [266]. DeepEnroll has outperformed the best baseline by up to 12.4% in an averaged-F1 score. In addressing the potential side effects of multi-drug combination administration (polypharmacy), Zitnik et al. (2018) presented a graph-embedding-based approach—Decagon—which builds multimodal graphs of protein–protein interactions and drug–protein target interactions and the polypharmacy side effects to model each relationship with nodes (i.e., drugs, proteins) and labeled edges (i.e., side effects) for multi-relational link prediction [265]. It was found that Decagon can accurately predict polypharmacy side effects, outperforming baselines by up to 69%. In a similar avenue of research focusing on drug-target interactions, DeepDTA, a convolutional neural networks (CNN)-based approach, was proposed to predict drug–target binding affinity using only sequences of proteins and drugs in a 1D representational state in the CNN model [264]. It has outperformed two state-of-the-art methods for DT binding affinity prediction, KronRLS algorithm and SimBoost, based on the concordance index (CI) to measure the model performance.

## 5. Conclusions

The task of finding a successful, novel drug as treatment for common diseases is predominantly a daunting yet arduous process, which is even more challenging for a rare genetic neurological disorder such as SMA. Many research and development pharmaceutical companies and research institutions are hesitant to pursue the drug development for rare diseases due to the small market size, high cost, possibly low return and lack of information about the disease, drugs and corresponding drug targets. Recently, CADD approaches have shown promising potentials in facilitating the drug discovery process and may be able to overcome the limiting bottlenecks of its traditional counterparts. Along with the advances of the knowledge of computational biology and informatics database, the opportunities provided by drug repurposing cannot be underestimated. The interactions of a drug and a target is a critical point of drug discovery. This information aids to establish correlations between diseases and targets in order to determine the therapeutic effect of drugs on various diseases. Hence, the well-known drug–disease relationships that has been established using network biology will help accelerate the target identification and lead optimization process for pre-clinical drug development. Integrated with the domain-specific AI in the ‘chemical big data,’ the novel approach could potentially serve as a panacea by increasing the efficiency of certain aspects of the drug discovery process.

Despite the promising potential offered by CADD, there are several challenges, including the access of databases consisting all the approved drugs and their detailed profiles, in-depth knowledge of disease, particularly for multifaceted disease, among others [267] to capitalize the benefit of CADD in advancing the domain of drug research and development. In spite of the recent advocacy of ‘open science’ in the scientific community, proprietary databases still remain few and far between. In addition, errors can be found in publicly available data, such as drug structures and their chemical profiles, among others, leading to the inevitable failure of identifying lead targets accurately. Research that involved multidisciplinary fields may face the challenges of integrating the complex theories into practical applications. This could only become more profound when dealing with the governance of data quality such as missing, biased and inaccurate data. Demonstrating this, the lack of the structural data of promising ligand or drug hinders the identification of potential drug–target interaction. Additionally, the protein–protein interaction network for a less-studied disease may mislead the drug discovery process. Addressing these challenges are by no means a trivial endeavor; monumental efforts must be put forth to develop a standardized, generic CADD framework to complement the traditional approach of creating novel yet effective therapeutics for both common and uncommon diseases. In light of such call-to-action, the various techniques and methodologies examined in this study may serve as a precedent in establishing the cornerstone for the CADD framework.

## Figures and Tables

**Table 1:** Therapeutic approaches in spinal muscular atrophy: clinical trial stage.

Drug Name|Structure|DrugBank ID	Mechanism of Action	Route of Administration	Clinical Trial Stages	Effect of Drugs	Type of SMA	Sponsor
**Risdiplam** (RG7916) * 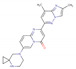 DrugBank ID: DB15305	*SMN2* splicing modifier	Oral (daily; through a g-tube)	II (JEWELFISH, RAINBOWFISH)	Two-fold increment in SMN protein concentration after 12 weeks of therapy	All types of SMA	Hoffmann-La Roche, PTC Therapeutics, SMA Foundation
**Branaplam** (LMI070, NVS-SM1) 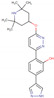 DrugBank ID: DB14918	*SMN2* splicing modifier	Oral	II	N/A	Type I	Novartis
**Reldesemtiv** (CK-2127107; 2-aminoalkyl-5-N-heteroarylpyrimidine) 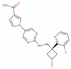 DrugBank ID: DB15256	Fast skeletal muscle troponin activator (FSTA)	Oral	II	Mild improvement in the six-minute walk test (6MWT) after 4 and 8 weeks of treatment	Type II/III/IV	Cytokinetics, Astellas
**SRK-015** ^#^	Myostatin inhibitor	Intravenous (IV) injection	II (TOPAZ)	Positive results in animal model	Type II/III	Scholar Rock

* Have been recently approved by FDA (as of August 2020) for the patients aged from 2 years old and above; however, it is still under evaluation for a broad range of patients. ^#^ No information of structure and DrugBank ID.

**Table 2:** List of databases that widely used for the *in silico* drug repurposing studies.

Category	Database	URL	Reference
Drug and target database	Binding Database (BindingDB)	https://www.bindingdb.org/bind/index.jsp (accessed on 29 December 2020)	[132,133]
	Biological General Repository for Interaction Datasets (BioGRID)	https://thebiogrid.org/ (accessed on 29 December 2020)	[134,135]
	ChEMBL	https://www.ebi.ac.uk/chembl/ (accessed on 29 December 2020)	[136,137]
	ChemDB	http://chemdb.ics.uci.edu/ (accessed on 29 December 2020)	[138]
	ChemSpider	http://www.chemspider.com/ (accessed on 29 December 2020)	[139]
	Connectivity Map (CMap)	https://portals.broadinstitute.org/cmap/ (accessed on 29 December 2020)	[140,141]
	Database of Interacting Proteins (DIP)	https://dip.doe-mbi.ucla.edu/dip/Main.cgi (accessed on 29 December 2020)	[142,143]
	Drug Repurposing Hub	https://clue.io/repurposing (accessed on 29 December 2020) (accessed on 29 December 2020)	[144]
	DrugBank	http://www.drugbank.ca/ (accessed on 29 December 2020)	[145]
	DrugCentral	http://drugcentral.org (accessed on 29 December 2020)	[146]
	Exascale Compound Activity Prediction Engine (ExCAPE-DB)	https://solr.ideaconsult.net/search/excape/ (accessed on 29 December 2020)	[147]
	GPS-Prot	http://gpsprot.org/ (accessed on 29 December 2020)	[148]
	Human Protein Reference Database (HPRD)	http://www.hprd.org/ (accessed on 29 December 2020)	[149,150]
	Library of Integrated Network-based Cellular Signatures (LINCS)	https://lincs.hms.harvard.edu/db/ (accessed on 29 December 2020)	[151]
	Molecular INTeraction database (MINT)	https://mint.bio.uniroma2.it/ (accessed on 29 December 2020)	[152,153]
	Proteopedia	http://proteopedia.org (accessed on 29 December 2020)	[154]
	PubChem	http://pubchem.ncbi.nlm.nih.gov (accessed on 29 December 2020)	[155]
	RCSB Protein Data Bank (PDB)	https://www.rcsb.org/ (accessed on 29 January 2021)	[156]
	Search Tool for the Retrieval of Interacting Genes/Proteins (STRING)	https://string-db.org/ (accessed on 29 December 2020)	[157]
	Structures of Well-curated Extracts, Existing Therapies, and Legally regulated Entities for Accelerated Discovery (SWEETLEAD)	https://simtk.org/projects/sweetlead (accessed on 29 December 2020)	[158]
	SuperDRUG2	http://cheminfo.charite.de/superdrug2/ (accessed on 29 December 2020)	[159]
	The NCGC Pharmaceutical Collection (NPC)	https://tripod.nih.gov/npc/ (accessed on 29 December 2020)	[160]
	The Universal Protein Resource (UniProt)	https://www.uniprot.org/ (accessed on 29 December 2020)	[161]
	ZINC	https://zinc.docking.org/ (accessed on 29 December 2020)	[162]
Pathway omics data	Kyoto Encyclopedia of Genes and Genomes (KEGG)	https://www.genome.jp/kegg/ (accessed on 29 December 2020)	[163,164]
	Mode of Action by NeTwoRk Analysis (MANTRA)	https://mantra.tigem.it/ (accessed on 29 December 2020)	[165,166]
	PathwayCommons	https://www.pathwaycommons.org/ (accessed on 29 December 2020)	[167,168]
	Reactome	https://reactome.org/ (accessed on 29 December 2020)	[169]
Genomics data	ArrayExpress	https://www.ebi.ac.uk/arrayexpress/ (accessed on 29 December 2020)	[170]
	GenBank	http://www.ncbi.nlm.nih.gov (accessed on 29 December 2020)	[171]
	Gene Expression Omnibus (NCBI-GEO)	http://www.ncbi.nlm.nih.gov/geo/ (accessed on 29 December 2020)	[172]
	Pharmacogene Variation (PharmVar)	https://www.pharmvar.org/ (accessed on 29 December 2020)	[173]
	Sequence Read Archive (SRA)	https://trace.ncbi.nlm.nih.gov/Traces/sra/ (accessed on 29 December 2020)	[174]
	Single Nucleotide Polymorphism database (dbSNP)	https://www.ncbi.nlm.nih.gov/snp/ (accessed on 29 December 2020)	[175]
Clinical and disease information	ClinicalTrials	https://clinicaltrials.gov/ (accessed on 29 December 2020)	-
	Comparative Toxicogenomics Database (CTD)	http://ctdbase.org/ (accessed on 29 December 2020)	[176]
	DisGeNET	https://www.disgenet.org/ (accessed on 29 December 2020)	[177]
	Drugs@FDA	https://www.accessdata.fda.gov/scripts/cder/daf/ (accessed on 29 December 2020)	-
	Genome-wide Association Studies (GWAS Catalog)	https://www.ebi.ac.uk/gwas/ (accessed on 29 December 2020)	[178]
	FDA Adverse Event Reporting System (FAERS)	https://open.fda.gov/data/faers/ (accessed on 29 December 2020)	[179]
	Online Mendelian in Man (OMIM)	https://www.ncbi.nlm.nih.gov/omim (accessed on 28 August 2020)	[180]
	OpenTargets	https://www.opentargets.org/ (accessed on 29 December 2020)	[181]
	Pharmacogenomics Knowledgebase (PharmGKB)	https://www.pharmgkb.org/ (accessed on 29 December 2020)	[182]
	Side Effect Resource (SIDER)	http://sideeffects.embl.de/ (accessed on 29 December 2020)	[183]
	Therapeutic Target Database (TTD)	http://db.idrblab.net/ttd/ (accessed on 29 December 2020)	[184]
Rare disease and orphan drugs	eRAM	http://www.unimd.org/eram/ (accessed on 29 December 2020)	[185]
	Orphanet (Oprhadata and Oprhanet Rare Disease Ontology (ORDO))	http://www.orpha.net (accessed on 29 December 2020)	-

**Table 3:** List of software tools for the *in silico* drug repurposing studies (neither extensive nor exhaustive) based on the respective approach with the additional required data.

Method	Approach	Required Data	Software Tools (Tool Name|Tool URL)	
Drug-oriented	*In silico* screening	Protein 3D structure, chemical structure, chemical information (targets and ligands)	**Protein structure prediction tools**	
			I-TASSER [199]	https://zhanglab.ccmb.med.umich.edu/I-TASSER/ (accessed on 29 December 2020)
			Modeller [200]	https://salilab.org/modeller/ (accessed on 29 December 2020)
			transform-restrained Rosetta [201]	http://robetta.bakerlab.org/ (accessed on 29 December 2020)
			**Docking**	
	Ligand based screening and molecular docking		AutoDock [202]	http://autodock.scripps.edu/ (accessed on 29 December 2020)
			AutoDock Vina [203]	http://vina.scripps.edu/ (accessed on 29 December 2020)
			High Ambiguity Driven protein-protein DOCKing (HADDOCK [204])	https://wenmr.science.uu.nl/haddock2.4/ (accessed on 29 December 2020)
			PatchDock [205]	https://bioinfo3d.cs.tau.ac.il/PatchDock/ (accessed on 29 December 2020)
			**Pharmacophore mapping and inverse virtual docking (IVD) programs**	
	Fragment-based screening		BIOVIA Discovery Studio	https://discover.3ds.com/discovery-studio-visualizer-download (accessed on 29 December 2020)
			INVDOCK [206]	http://bidd.group/group/softwares/invdock.htm (accessed on 29 December 2020)
			LigandScout [207]	http://www.inteligand.com/ligandscout/ (accessed on 29 December 2020)
			PharmMap	http://www.meilerlab.org/index.php/research/show?w_text_id=32 (accessed on 29 December 2020)
			PharmMapper [208,209]	http://www.lilab-ecust.cn/pharmmapper/ (accessed on 29 December 2020)
			ZINCPharmer [210]	http://zincpharmer.csb.pitt.edu/ (accessed on 29 December 2020)
	Drug similarity studies	Chemical structure, chemical information of drugs, clinical trial information, side effects and adverse events, FDA approval labels	**Drug-drug similarities prediction and visualization**	
			ChemMine Tools [211]	http://chemmine.ucr.edu/ (accessed on 29 December 2020)
			ChemTreeMap [212]	https://chemtreemap.readthedocs.io/en/latest/ (accessed on 29 December 2020)
			Compound Specific bioActivity DENdrogram (C-SPACE [213])	http://cspade.fimm.fi/ (accessed on 29 December 2020)
			**Drug-drug similarities and drug-target interaction prediction**	
			SuperPred [214]	https://prediction.charite.de/ (accessed on 29 December 2020)
Disease-/therapy-oriented	Signature-based drug repurposing	Gene signatures information, disease/genetics data, drug omics data	**Signature-based drug repurposing tool**	
			Cogena [215]	http://bioconductor.org/packages/release/bioc/html/cogena.html (accessed on 29 December 2020)
			ksRepo [216]	https://github.com/adam-sam-brown/ksRepo (accessed on 29 December 2020)
			DrugSig [217]	http://biotechlab.fudan.edu.cn/database/drugsig/ (accessed on 29 December 2020)
	Pathway-/network-based drug repurposing	General drug information, pathway information	**Network-based drug repurposing tool**	
			Drug Repurposing Recommendation System (DRRS [218])	http://bioinformatics.csu.edu.cn/resources/softs/DrugRepositioning/DRRS/index.html (accessed on 29 December 2020)
			DrugNet [219]	http://genome.ugr.es:9000/drugnet (accessed on 29 December 2020)
			GeneDiseaseRepositioning [220]	https://bitbucket.org/ncl-intbio/genediseaserepositioning/src/master/ (accessed on 29 December 2020)
			Predicting Drugs having Opposite effects on Disease genes (PDOD [221])	http://gto.kaist.ac.kr/pdod/index.php/main (accessed on 29 December 2020)
	Targeted mechanism-based drug repurposing		**Network visualization**	
			Cytoscape [222]	https://cytoscape.org/ (accessed on 29 December 2020)
			GeneMANIA [223]	https://genemania.org/ (accessed on 29 December 2020)
			Pathway Studio [224]	https://www.pathwaystudio.com/ (accessed on 29 December 2020)
			PATIKAweb [225]	http://www.cs.bilkent.edu.tr/~patikaweb/ (accessed on 29 December 2020)
			VisANT [226]	http://www.visantnet.org/visantnet.html (accessed on 29 December 2020)

**Table 4:** Utility of machine learning methods in addressing the challenges of drug development.

Machine Learning Methods	Area of Drug Development	Reference
Generative model Reinforcement learning	*Design and Discovery and Preclinical Research* Synthesis prediction and de novo drug design Virtual drug screening and drug target identification	[261,262,263]
Deep representational learning Graph embeddings	*Clinical Research and Safety Monitoring* Clinical trial recruitment Adverse drug effects, polypharmacy and drug-food interaction prediction	[264,265,266]

## Abbreviations

SMA	spinal muscular atrophy
SMN1	survival motor neuron 1
FDA	U.S. Food and Drug Administration
NMD	neuromuscular disease
AI	artificial intelligence
ML	machine learning
DL	deep learning
ALS	amyotrophic lateral sclerosis
R&D	research and development
α-MNs	alpha motor neurons
FL-SMN	full length, functional SMN
OMIM	Online Mendelian Inheritance in Man
PDB	Protein Data Bank
Ge2BD	Gemin2 binding domain
snRNP	spliceosomal small nuclear ribonucleoprotein
sDMA	symmetric dimethylarginine
ASO	antisense oligonucleotide
CNS	central nervous system
AAV9	adeno-associated virus 9
cDNA	complementary DNA
hnRNP	heterogenous nuclear ribonucleoprotein
FSTA	fast skeletal muscle troponin activator
BBB	blood–brain barrier
6MWT	six-minute walk test
MEP	maximal expiratory pressure
CADD	computer-aided drug design
ADME	absorption, distribution, metabolism and extraction
HTS	high throughput screening
NDD	network-driven drug discovery
AID	artificial intelligence (AI)-assisted drug discovery
HDAC	Histone deacetylase inhibitors
VPA	valproic acid
SMILES	Simplified Molecular Input Line Entry System
InChl	International Chemical Identifier
Tc	Tanimoto coefficient
DTIs	drug-target interactions
NCBI	National Center for Biotechnology Information
GEO	Gene Expression Omnibus
dbSNP	Single Nucleotide Polymorphism database
SRA	Sequence Read Archive
ADEs	adverse drug events
SIDER	side effect resource
CMap	connectivity map
NIH	National Institutes of Health
LINCS	library of integrated network based cellular signatures
KEGG	Kyoto Encyclopedia of Genes and Genomes
ROCK	RhoA/Rho kinase
cAMP	cyclic adenosine monophosphate
ERK	extracellular regulated kinase
JNK	c-Jun N-terminal Kinase
GPUs	graphical processing units
TPUs	tensor processing units
QSAR	quantitative structure-activity relationship
OCD	obsessive compulsive disorder
RF	random forests
SVM	support vector machines
NN	neural networks
GAN	generative adversarial neural network
BERT	Bidirectional Encoder Representations from Transformers
CNN	convolutional neural networks
CI	concordance index
